# Effects of EEG burst suppression on cerebral oxygen metabolism and postoperative cognitive function in elderly surgical patients: A randomized clinical trial

**DOI:** 10.1097/MD.0000000000033148

**Published:** 2023-03-31

**Authors:** Min Liu, Qi-Qi Wang, Wen-Xin Lin, Bao-Xin Ma, Quan-Yang Lin

**Affiliations:** a Department of Anesthesiology, Zhongshan Hospital Xiamen University, Xiamen, China; b Department of Anesthesiology, Women and Children’s Hospital Xiamen University, Xiamen, China.

**Keywords:** burst suppression, cerebral oxygen metabolism, cognitive function, electroencephalogram

## Abstract

This randomized clinical trial determined the effects of electroencephalographic burst suppression on cerebral oxygen metabolism and postoperative cognitive function in elderly surgical patients.

**Methods:**

The patients were placed into burst suppression (BS) and non-burst suppression (NBS) groups. All patients were under bispectral index monitoring of an etomidate target-controlled infusion for anesthesia induction and intraoperative combination sevoflurane and remifentanil for anesthesia maintenance. The cerebral oxygen extraction ratio (CERO_2_), jugular bulb venous saturation (SjvO_2_), and difference in arteriovenous oxygen (Da-jvO_2_) were measured at *T*0, *T*1, and *T*2. One day before surgery, and 1, 3, and 7 days after surgery, postoperative cognitive dysfunction was assessed using the mini-mental state examination (MMSE).

**Results:**

Compared with *T*0, the Da-jvO_2_ and CERO_2_ values were decreased, and SjvO_2_ was increased in the 2 groups at *T*1 and *T*2 (*P* < .05). There was no statistical difference in the SjvO_2_, Da-jvO_2_, and CERO_2_ values between *T*1 and *T*2. Compared with the NBS group, the SjvO_2_ value increased, and the Da-jvO_2_ and CERO_2_ values decreased at *T*1 and *T*2 in the BS group (*P* < .05). The MMSE scores on the 1st and 3rd days postoperatively were significantly lower in the 2 groups compared to the preoperative MMSE scores (*P* < .05). The MMSE scores of the NBS group were higher than the BS group on the 1st and 3rd days postoperatively (*P* < .05).

**Conclusion:**

In elderly patients undergoing surgery, intraoperative BS significantly reduced cerebral oxygen metabolism, which temporarily affected postoperative neurocognitive function.

## 1. Introduction

As an electroencephalographic (EEG) modality, burst suppression consists of alternating periods of high-amplitude slow waves (bursts) and so-called flat EEG (suppression) periods.^[1]^ Burst suppression on an EEG is defined as a suppression period exceeding 0.5 seconds with an amplitude of ≤ 5 μV in humans.^[2]^ Burst suppression on EEG is an EEG pattern in which low-voltage “suppression” alternates with high-voltage “bursts.” Purposeful application of general anesthetics to induce burst suppression increases brain tissue tolerance to the degree and time of subsequent ischemia-hypoxic injury, and achieve the purpose of brain protection.^[3,4]^

A lower percentage of operative time spent with EEG suppression was related to processing the EEG to maintain a Patient State Index > 35, and a higher percentage of operative time was related to preoperative cognitive impairment.^[5]^ A previous study showed that burst suppression may be neuroprotective in acute patients with traumatic brain injury etiologies.^[6]^ As surgery rates increase, so does the number of elderly patients with postoperative cognitive dysfunction (POCD).^[7]^ The degree of intraoperative EEG suppression and low regional cerebral oxygen saturation at the end of surgery are related to POCD in patients with cardiac intervention.^[8]^ Monitoring burst suppression may have clinical utility, especially in patients with cognitive impairment. Intraoperative EEG monitoring may be used as a method to guide anesthesia management.^[9]^ A previous study indicated that EEG burst suppression is related to propofol bolus, younger age, inhalation anesthetic, and lower arterial pressure.^[10]^ It has not been established if burst suppression has a cerebral protective effect in elderly surgical patients.

The aim of this study was to determine the effect of burst suppression on cerebral oxygen metabolism and postoperative cognitive function in elderly surgical patients to evaluate the cerebral protective effect.

## 2. Materials and methods

### 2.1. Patient information

This was a randomized consort trial. The study was approved by the Ethics Committee of Zhongshan Hospital Xiamen University (No. xmzsyyky-ethics-2017-033) on February 27, 2017. Informed written consent was obtained from each patient and his/her family members.

The inclusion criteria were as follows: age > 65 years; operative time ≤ 3 hours; body mass index < 30 kg/m^2^; and American Society of Anesthesiologists grade I to III. Patients with factors that may lead to abnormal brain electrical activity were excluded, including a preoperative history of mental illness, an abnormal EEG, use of antiepileptic and antipsychotic drugs, acid-base balance metabolic disorders, water and electrolyte imbalance, alcoholism, hypoxemia, carbon dioxide storage, diabetes mellitus, a history of lung surgery, and a history of allergy to etomidate and other research drugs.

**Figure 1.**
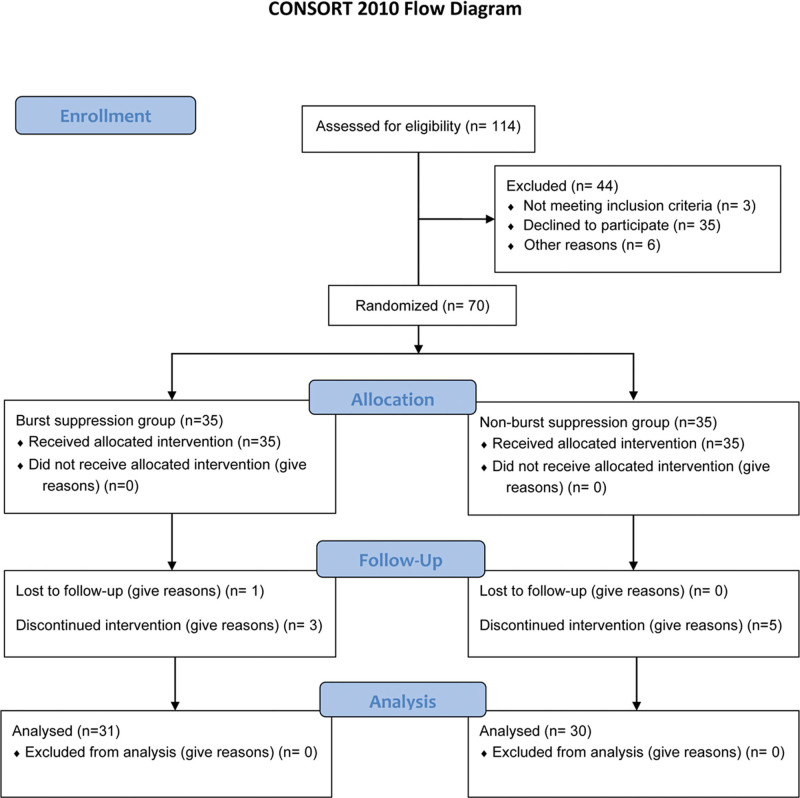
Flow diagram of patient enrollment.

One hundred fourteen elderly patients who underwent elective general anesthesia (all types) were assessed for eligibility. According to the inclusion and exclusion criteria, 70 patients were enrolled in the study (Fig. 1). The randomly-generated group information was placed in an opaque, sealed envelope by an investigator and given to the subject by an uninformed person. The experimental operator used the group information for experimental and invasive procedures, and another researcher was responsible for postoperative and follow-up evaluations. Each aspect of the study was kept confidential to ensure blindness. According to the random method, all patients were randomly classified into (burst suppression [BS]; n = 35) and (non-burst suppression [NBS]; n = 35) groups at a 1:1 ratio. One patient with an inaccurate position of the venous catheter, 1 patient with a burst suppression < 2 or who could not be induced continuously for 30 minutes, 1 patient with an inaccurate blood gas analysis, and 1 patient who did not cooperate with the evaluation were excluded from the BS group; 31 patients were included in the final analysis. Two patients with arterial and venous puncture failure and inaccurate catheter locations, 2 patients with inaccurate blood gas analyses, including air mixing, and 1 patient with burst suppression that occurred spontaneously during routine anesthesia were excluded; 30 patients were included in the final analyses.

### 2.2. Anesthesia process

Preoperative medications were not used in any patient. After entering the operating room, upper extremity venous access was established, 8 to 10 mL·kg^-1^·hour^-1^ of sodium lactate Ringer solution was infused, and an electrocardiogram, heart rate (HR), noninvasive blood pressure, and SpO_2_ were monitored continuously. According to the instructions, disposable EEG sensor electrodes were pasted on the patient’s forehead, then connected to the bispectral index (BIS) Vista monitor (Covidien, USA) to monitor BIS and burst suppression. The measured resistance met the monitoring requirements (<5 kΩ), and various electromagnetic interference sources were maintained at a safe distance. The operating room was then kept quiet and the patient’s body temperature was maintained. An invasive arterial puncture was performed under local anesthesia with lidocaine after demonstrating a negative Allen test. After a successful puncture, the pressure measuring device was connected to a fixed cannula, and 2 mL of blood was used for blood gas analysis and dynamic monitoring of noninvasive blood pressure. The patient was placed in a slightly head-down position, and the distance from the mastoid to the puncture point was measured and recorded as the depth of catheter placement for Jugular bulb puncture and catheterization. After sterilizing and laying a towel, lidocaine was punctured cephalad through the right internal jugular vein under local anesthesia and a catheter was placed in a retrograde fashion until the jugular bulb encountered resistance. Then the catheter was withdrawn 0.5 to 1 cm and fixed. Two milliliters of blood was collected through the catheter, the blood collection speed was < 2 mL/minutes, and the blood gas analysis of the internal (jugular bulb venous saturation [SjvO_2_], 55% to 75%) was carried out. After confirming the correct position, the catheter was fixed, and the indwelling catheter was slowly infused at a rate of 3 mL/minutes. Etomidate target-controlled infusion was started with a target-controlled infusion-III syringe pump for intravenous induction after mask inhalation of pure oxygen. The pharmacokinetics followed the Arden model. The initial target plasma concentration was set to 0.8 μg/mL. After the BIS value was < 50, rocuronium (0.6 mg/kg) and sufentanil (0.3 μg/kg) were administered in sequence, then tracheal intubation was performed to control breathing and maintain an end-tidal carbon dioxide partial pressure of 35 to 40 mm Hg (1 mm Hg = 0.133 kPa). Continuous infusion of remifentanil (0.2 to 0.3 μg/kg/minutes) combined with inhalation of 1% to 1.5% sevoflurane and intermittent intravenous rocuronium bromide was used for maintenance of anesthesia. After induction, the plasma target concentration of etomidate was increased to 1.4 μg/mL in the BS group, and gradually increased with a dose gradient of 0.1 μg/mL. Each target concentration was maintained for 10 minutes until burst suppression appeared. The criteria for burst suppression were as follows: burst suppression rate (BSR) > 10%; duration > 1 minute^[11]^; adjust the plasma target control concentration of etomidate according to the BSR; and maintain BS for at least 30 minutes. The plasma target concentration of etomidate was used for maintenance of the BIS value (40–60) until the operation was completed in the NBS group. The following actions were taken, as indicated: atropine 0.5 mg was administered intravenously for an HR < 45/minutes; fluid replacement was accelerated and ephedrine (10 mg) was injected intravenously when the mean arterial pressure decreased by > 30% of the baseline value; intravenous injection of esmolol (10 mg) was administered for an HR > 100/minutes; and intravenous urapidil (15 mg) was administered and repeated if necessary when the mean arterial pressure increased > 30% of the baseline value.

### 2.3. Detection of cerebral oxygen metabolism indices in patients

Arterial and internal jugular vein bulb blood were drawn, and blood gas analyses were performed at *T*0 (before anesthesia), *T*1 (BSR > 2 occurs and maintained for 1 minute in the BS group and after etomidate target-controlled infusion for 8 minutes in the NBS group), and *T*2 (BSR > 2 occurs and maintained for 30 minutes in the BS group and after etomidate target-controlled infusion for 38 minutes in the NBS group). The primary endpoints were the cerebral oxygen extraction ratio (CERO_2_), SjvO_2_, and difference in arteriovenous oxygen (Da-jvO_2_), all of which were recorded and analyzed. The CERO2, SjvO2, and Da-jvO_2_ were obtained according to the Fick formula, as follows:

CaO_2_ = 1.36 × hemoglobin × SaO_2_ + 0.0031 × PaO_2_; CERO_2_ = (CaO_2_-CjvO_2_)/CaO_2_ × 100%; SjvO_2_ = 1.36 × hemoglobin × SjvO_2_ + 0.0031 × PjvO_2_; and Da-jvO_2_ = CaO_2_-CjvO_2_.

### 2.4. Mini-mental state examination (MMSE) score

As a secondary endpoint for this study, the MMSE was applied to assess POCD according to a previous study.^[12]^ The MMSE was administered 1 day before surgery and on the 1st, 3rd, and 7th days after surgery.

### 2.5. Statistical analysis

SPSS 24.0 (BM Corporation, New Orchard Road, Armonk, NY) was used for data analysis. The measurement data are expressed as the mean ± standard deviation ($\bar{x}\pm s$). A paired *t* test was used for comparisons within groups, repeated measures analysis of variance was applied for comparison between groups, and a chi-square test was applied for comparison of counting data. A *P* < .05 was considered to be a significant difference.

## 3. Results

### 3.1. Basic information

There were no differences in gender ratio, age, American Society of Anesthesiologists grade, body mass index, and operative time between the NBS and BS groups (*P* > .05; Table 1).

**Table 1:** Comparison of general conditions of the 2 groups of patients.

Groups	BS (N = 26)	NBS (N = 27)	*P* value
Gender (M/F)	12/14	12/15	.901
Age (yr)	74 ± 5	73 ± 6	.891
ASA grading(I/II/III)	6/12/8	7/13/7	.486
Operation time (min)	112 ± 8	115 ± 6	.802
BMI (kg/m^2^)	23.9 ± 2.9	23.2 ± 2.6	.641

ASA = American Society of Anesthesiologists, BMI = body mass index, BS = burst suppression, NBS = non-burst suppression.

### 3.2. Comparison of cerebral oxygen metabolism indices between the 2 groups

**Figure 2.**
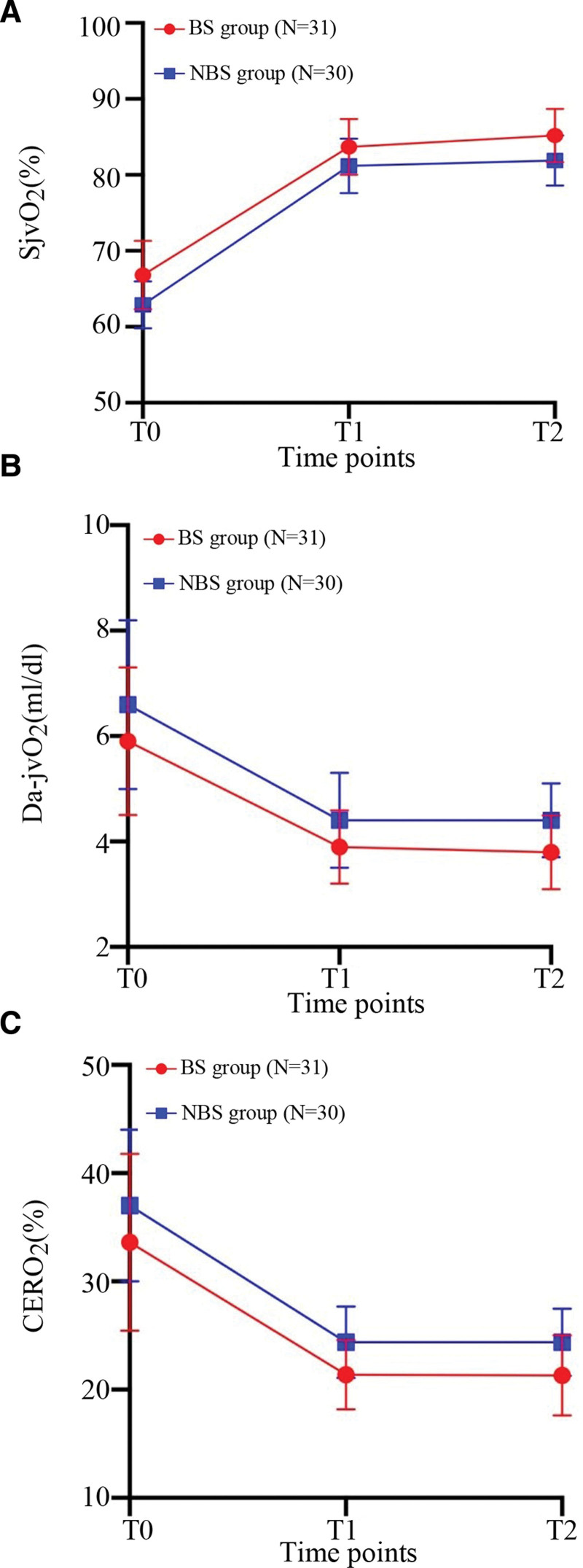
Comparison of SjvO_2_ (A), Da-jvO_2_ (B), and CERO_2_ (C) at each time point between the 2 groups. CERO2 = cerebral oxygen extraction ratio, Da-jvO2 = difference of arteriovnous oxygen, SjvO2 = jugular bulb venous saturation.

Compared with *T*0, the Da-jvO_2_ and CERO_2_ values were decreased at *T*1 and *T*2, and the SjvO_2_ value was increased (*P* < .05). The SjvO_2_, Da-jvO_2_, and CERO_2_ values showed no differences between *T*1 and *T*2 (*P* > .05). Compared with the BS group, the SjvO_2_ value was decreased at *T*1 and *T*2 in the NBS group, and the Da-jvO_2_ and CERO_2_ values were increased significantly (Table 2 and Fig. 2).

**Table 2:** Comparison of cerebral oxygen metabolism indexes between the 2 groups at each time point.

Indicators	SjvO_2_ (%)			Da-jvO_2_ (mL/dL)			CERO_2_ (%)		
	BS	NBS	*P* value	BS	NBS	*P* value	BS	NBS	*P* value
*T*_0_	66.8 ± 4.5	65.9 ± 3.1	.866	5.9 ± 1.4	6.6 ± 1.6	.872	33.6 ± 8.2	37.0 ± 7.0	.182
*T*_1_	83.7 ± 3.7*	76.2 ± 3.6*,†	.028	3.5 ± 0.7*	4.4 ± 0.9*,†	.001	20.4 ± 3.2*	24.4 ± 3.3*,†	.001
*T*_2_	85.2 ± 3.5*	78.9 ± 3.3*,†	.027	3.5 ± 0.7*	4.4 ± 0.7*,†	.001	20.3 ± 3.7*	24.4 ± 3.1*,†	.002

Compared with *T*0.

BS = burst suppression, CERO_2_ = cerebral oxygen extraction ratio, Da-jvO_2_ = difference of arteriovnous oxygen, NBS = non-burst suppression, SjvO_2_ = jugular bulb venous saturation.

* *P* < .05, compared with NBS group.

† *P* < .05.

### 3.3. Comparison of MMSE scores between the NBS and BS groups

Compared with the preoperative MMSE scores, the MMSE scores of the 2 groups on the 1st and 3rd days postoperatively were significantly decreased (*P* < .05). The MMSE scores of the BS group were significantly lower than the NBS group on the 1st and 3rd days postoperatively (*P* < .05; Table 3).

**Table 3:** Comparison of perioperative MMSE scores between the 2 groups (N = 30).

Times	BS	NBS	*P* value
Preoperative	24.5 ± 3.2	25.2 ± 3.8	.922
Day 1 after surgery	21.8 ± 4.1*,†	23.1 ± 4.2*	.007
Day 3 after surgery	22.8 ± 3.7*,†	24.4 ± 4.0*	.001
Day 7 after surgery	24.1 ± 3.6	24.9 ± 4.3	.781

compared with *T*0.

BS = burst suppression; MMSE = Mini-Mental State Examination, NBS = non-burst suppression.

* *P* < .05, compared with NBS group.

† *P* < .05.

## 4. Discussion

BS is an EEG pattern which is caused by various etiologies and has a poor outcome.^[13]^ BS does not occur during physiologic sleep,^[14]^ rather BS is reversibly induced by administration of anesthetics and/or by cooling the brain.^[15]^ Actively-induced BS in the perioperative period has a protective effect on the brain. Most of the commonly-used anesthetics induce BS. Sevoflurane had a safer profile on cerebral oxygenation during BS.^[16]^ BS shows that sevoflurane exerts an intrinsic cerebral vasodilatory effect.^[17]^ The subjects in the current study were elderly, thus etomidate combined with sevoflurane anesthesia was used.^[18,19]^ Both drugs have the advantage of circulatory stability, which can reduce the impact on the circulation while maintaining BS, and reduce the dosage and side effects of both drugs.

Oxygen consumption and supply in the brain tissues are considered to be important indicators of normal aerobic metabolism in the brain. Indeed, monitoring the supply and demand balance of cerebral oxygen during surgical anesthesia is helps protect the brain.^[20]^ SjvO_2_ and CERO_2_ are the key indices for monitoring cerebral oxygen metabolism.^[7,21]^ In the current study, the SjvO_2_ value was shown to increase with the prolongation of anesthesia time after anesthesia induction. The normal value of SjvO_2_ is 55% to 75% on room air. In the current study, the SjvO_2_ value of patients intraoperatively was significantly higher compared with the SjvO_2_ value before anesthesia due to improvement with pure oxygen inhalation and ventilation. In addition, the CERO_2_ and Da-jvO_2_ values of patients decreased significantly after anesthesia, indicating that etomidate combined with sevoflurane, like most intravenous gamma-aminobutyric acid receptor drugs, reduces the cerebral oxygen uptake rate. At *T*1 and *T*2, the CERO_2_ value in the NBS group was significantly increased compared to the BS group, likely because BS represents the depth of anesthesia and inhibits the brain activity at deeper level, thus resulting in a more apparent decrease in cerebral oxygen metabolism. Compared with the CERO_2_ and Da-jvO_2_ values, there was no difference between *T*1 and *T*2 in the BS group, indicating that increasing the time after BS does not further reduce cerebral oxygen metabolism. This finding verified the basic neurometabolic mechanism of burst inhibition proposed by Ching et al^[22]^ This mechanism considers BS as a means for cell survival by allocating maximum energy to essential cellular functions, thereby preventing collapse of the membrane potential in a low metabolic state.

The findings of Tang et al^[5]^ suggested that POCD is reduced by interventions that reduce EEG BS. In this study the MMSE scores of the 2 groups of patients on the 1st and 3rd days postoperatively were significantly lower than preoperatively, and returned to the preoperative score on the 7th day postoperatively. The MMSE scores of the BS group on the 1st and 3rd days postoperatively were lower than the NBS group. Our results suggested that anesthesia surgery factors affect the postoperative cognitive ability of elderly patients, similar to the results of previous studies.^[23–25]^ Our results also indicated that intraoperative EEG BS may increase the incidence of POCD. In a study of elderly patients > 65 years of age, the EEG amplitude and maximum EEG slope decreased significantly with age, which corresponds well to the correlation between intraoperative BS and POCD.^[26]^ On the 7th day postoperatively, the MMSE scores were not different in the 2 groups and were similar to the preoperative scores, indicating that the postoperative neurocognitive dysfunction caused by BS can be recovered in a short time. In our study it was also observed that patients with low preoperative MMSE scores were more likely to have BS and a longer duration of BS than other patients. Therefore, BS may be an indicator of susceptibility to POCD.

There were some limitations in this study. First, our results were based on a small sample; the results should be verified in a larger population. Second, the cerebral protective mechanism is still not clear, and further research is needed.

## 5. Conclusion

Intraoperative BS increases the SjvO_2_ value and decreases the CERO_2_ and Da-jvO_2_ values, which has a cerebral protective effect and can temporarily reduce postoperative neurocognitive function in elderly surgical patients. More attention should be paid to intraoperative BS in clinical practice.

## Author contributions

**Conceptualization:** Min Liu, Quan-Yang Lin.

**Data curation:** Min Liu, Qi-Qi Wang, Bao-Xin Ma, Quan-Yang Lin.

**Formal analysis:** Qi-Qi Wang, Wen-Xin Lin, Bao-Xin Ma.

**Methodology:** Wen-Xin Lin.

**Writing – original draft:** Min Liu, Quan-Yang Lin, Qi-Qi Wang.

**Writing – review & editing:** Quan-Yang Lin.
